# Long-term prednisone treatment causes fungal microbiota dysbiosis and alters the ecological interaction between gut mycobiome and bacteriome in rats

**DOI:** 10.3389/fmicb.2023.1112767

**Published:** 2023-06-05

**Authors:** Wenyan Li, Yun Shu, Jing Zhang, Mengmeng Wu, Guang-hua Zhu, Wen-yan Huang, Li Shen, Yulin Kang

**Affiliations:** ^1^Department of Nephrology and Rheumatology, Shanghai Children’s Hospital, School of Medicine, Shanghai Jiao Tong University, Shanghai, China; ^2^Department of Cardiothoracic Surgery, Shanghai Children’s Hospital, School of Medicine, Shanghai Jiao Tong University, Shanghai, China

**Keywords:** glucocorticoid, gut fungi, bacteria, fecal metabolites, short chain fatty acids

## Abstract

Glucocorticoids (GCs) are widely used in the treatment of immune-mediated diseases due to their anti-inflammatory and immunosuppressive effects. Prednisone is one of the most commonly used GCs. However, it is still unknown whether prednisone affects gut fungi in rats. Herein we investigated whether prednisone changed the composition of gut fungi and the interactions between gut mycobiome and bacteriome/fecal metabolome in rats. Twelve male Sprague–Dawley rats were randomly assigned to a control group and a prednisone group which received prednisone daily by gavage for 6 weeks. ITS2 rRNA gene sequencing of fecal samples was performed to identify differentially abundant gut fungi. The associations between gut mycobiome and bacterial genera/fecal metabolites obtained from our previously published study were explored by using Spearman correlation analysis. Our data showed that there were no changes in the richness of gut mycobiome in rats after prednisone treatment, but the diversity increased significantly. The relative abundance of genera *Triangularia* and *Ciliophora* decreased significantly. At the species level, the relative abundance of *Aspergillus glabripes* increased significantly, while *Triangularia mangenotii* and *Ciliophora* sp. decreased. In addition, prednisone altered the gut fungi-bacteria interkingdom interactions in rats after prednisone treatment. Additionally, the genus *Triangularia* was negatively correlated with m-aminobenzoic acid, but positively correlated with hydrocinnamic acid and valeric acid. *Ciliophora* was negatively correlated with phenylalanine and homovanillic acid, but positively correlated with 2-Phenylpropionate, hydrocinnamic acid, propionic acid, valeric acid, isobutyric acid, and isovaleric acid. In conclusion, long-term prednisone treatment caused fungal microbiota dysbiosis and might alter the ecological interaction between gut mycobiome and bacteriome in rats.

## Introduction

Glucocorticoids (GCs) are widely used in the treatment of immune-mediated diseases and organ transplantation due to their anti-inflammatory and immunosuppressive effects (Vandewalle et al., 2018). Prednisone is one of the most commonly used GCs. In the liver, prednisone is converted by 11-hydroxylation to the active metabolite prednisolone (Jeries et al., 2020). Long-term prednisone treatment leads to a number of side effects including obesity, growth retardation, hypertension and hyperglycaemia (Seale and Compton, 1986). The efficacy of GCs is mediated by genomic and non-genomic mechanisms (Vandewalle et al., 2018). The genomic mechanism works through the GC receptor (GR) in cells. The GC diffuses across the cell membrane and binds to the GC receptor (GR) in the cytoplasm. The GC/GR complex is then translocated to the nucleus and binds to the GC response elements (GREs) of DNA, thereby reducing the expression of inflammatory factors (Adcock, 2000). The non-genomic mechanism is independent of nuclear gene transcription, which has not been as extensively studied as the genomic effects.

Our previous published study aimed to explore the non-genomic mechanism in terms of the gut microbiota. It shows that prednisone changes the composition of gut bacteria. The relative abundances of the genera *Eisenbergiella*, *Alistipes*, and *Clostridium XIVb* decrease significantly, while *Anaerobacterium* increases. Prednisone also alters the fecal metabolome, which may be involved in the pharmacology of GCs (Zhang et al., 2021). However, gut bacteria are only part of gut microbiota in host. It will be useful to investigate the effect of GCs on gut fungi which are an underestimated part of microbial community.

Gut microbiota is a highly complex microbial ecosystem that includes bacteria, fungi, archaea, and viruses (Mills et al., 2019). The structure of enteric microbiota can be influenced by drugs, diet, age, gender, race, environment, and genetic background of the host (Zhang et al., 2015; Capurso, 2016; Yamada et al., 2016).The metabolites of gut microbes can affect the host health. For example, the proportion of butyrate-producing bacteria decreases significantly in children with steroid-dependent nephrotic syndrome, but it increases when the disease is relieved (Tsuji et al., 2018). Some toxins produced by gut microbes, including indole, ammonia and trimethylamine oxide, promote the progression of chronic kidney disease in host (Vaziri et al., 2016).

Fungi are an important part of gut microbiota (Belvoncikova et al., 2022). Recent metagenomic sequencing revealed that human gut fungal community comprises less than 0.1% of the microbes in the gut ecosystem, but changes in gut fungal composition are associated with a number of diseases such as inflammatory bowel disease (IBD), irritable bowel syndrome and colorectal cancer (Mukherjee et al., 2015; Coker et al., 2019; Richard and Sokol, 2019; Fiers et al., 2020). Gut mycobiome is involved in the gut-brain axis and host immunity (Carabotti et al., 2015; Iliev and Leonardi, 2017). Furthermore, there are the interactions between gut mycobiome and bacteriome (Hoarau et al., 2016). It is known that antifungal medications not only reduce the diversity of gut mycobiome, but also promote the growth of pathogenic bacteria (Qiu et al., 2015). Similarly, broad-spectrum antibiotics affect fungal colonisation (Samonis et al., 1993). However, it is still unknown whether prednisone affects gut fungi in rats, which are the commonly used rodents to study immune-mediated diseases.

In this study, we investigated the effect of long-term prednisone treatment on the composition of gut fungi and the interaction between gut mycobiome and bacteriome/fecal metabolome in rats.

## Materials and methods

### Animals and experimental design

The animal experiment was approved by the Ethical Committee of Shanghai Children’s Hospital, School of Medicine, Shanghai Jiao Tong University. The samples for this experiment were obtained from our previous research project (Zhang et al., 2021). Briefly, we collected 12 fecal samples from specific pathogen-free grade male Sprague–Dawley (SD) rats (Jiesijie Experimental Animal Company, Shanghai, China, 5 weeks old), which were randomly assigned to control (CON) and prednisone (PRED) groups after 10 days of habituation. All rats were housed under standard conditions with optimal temperature (22–25°C), relative humidity (50–60%), light/dark cycle (12: 12 h), food and water *ad libitum*. The 10 mg/kg of prednisone (Xinyi Pharmacy Company, Shanghai, China) dissolved in water was administered daily by gavage to each rat in the PRED group for 6 weeks, while the same amount of water was administered to rats in the CON group as previously described (Xu et al., 2016). Fecal pellets discharged by rats were collected after 6 weeks treatment.

### Fecal mycobiome profiling

Total DNA was extracted using QIAampDNA fecal kit (Qiagen, Hilden, Germany), and the integrity was assessed by agarose gel electrophoresis. The library was constructed by polymerase chain reaction (PCR) to amplify the ITS2 rRNA gene of fungus. The forward primer 5’-GCATCGATGAAGAACGCAGC-3′ and reverse primer 5’-TCCTCCGCTTATTGATATGC-3′ were used in this study. The Gene Amp PCR-System 9,700 (American Applied Biological Systems Company, United States) was used for PCR, with a total volume of 50 μL, including 10 μL of 5 × PCR buffer, 1 μL dNTP, 1 unit Phusion DNA Polymerase, 2 μL primers, and 50 ng template DNA. The thermal cycling conditions were as follows: initial denaturation at 94°C for 2 min, then 30 cycles of denaturation at 94°C for 30 s, annealing at 50°C for 30 s, extension at 72°C for 30 s, and finally 5 min at 72°C for extension. The quality of the amplified products was examined by gel electrophoresis. All PCR products were extracted by using the AxyPrepDNA gel extraction kit (AXYGEN Company, United States) and quantified busing the FTC-3000™ real-time PCR instrument (Funglyn Biotech Company, Canada). The samples were then amplified by the secondary PCR. The total volume of the secondary PCR was 40 μL, including 8 μL of 5 × PCR buffer, 1 μL dNTP, 0.8 unit Phusion DNA Polymerase, 2μL outer primers, and 5 μL template DNA. The thermal cycling conditions were as follows: initial denaturation at 94°C for 2 min, 8 cycles at 94°C for 30 s, 56°C for 30 s, 72°C for 30 s, and finally 5 min at 72°C. The amplifiers of ITS2 rRNA gene were sequenced on the Illumina MiSeq platform from Shanghai Tiny Gene Technology Company.

### Bioinformatic analysis

The operational taxon (OTU) is clustered with 97% similarity cutoff value through the UPARSE channel. The OTUs were classified according to the ribosomal database project (RDP). The R (version:3.6.0) vegan (version:2.5-5) and picante (version:1.8) software were used to calculate alpha diversity to evaluate the microbial richness and diversity, including Chao, ACE, Simpson and PD_whole_tree index. The R (version:3.6.0) vegan (version:2.5-5) was used to calculate beta diversity, including Bray-Curtis, Jaccard, unweighted UniFrac and weighted UniFrac distance. Linear discriminant analysis (LDA) effect size analysis (LEfSe) was used to identify differentially abundant gut fungi in rats between two groups at the taxonomic levels. Bioinformatics analysis was performed by Tiny Gene Technology Company (Shanghai, China).

For the re-analysis of the data of gut bacteria and fecal metabolites, the bioinformatics methods were the same as previously described (Zhang et al., 2021). Briefly, differential abundant gut bacteria at taxonomic levels were identified by using the Metastats method. The identification of differential fecal metabolites was based on volcano plot (*p* < 0.05) and variable influence on projection analysis (VIP analysis) (VIP > 1.0, *p* < 0.05). The associations between gut mycobiome and bacterial genera, gut mycobiome and fecal metabolites were determined by using Spearman correlation analysis and performed with R version 3.6.3. The differential abundant gut bacteria/fungus and differential fecal metabolites were included in this correlation analysis. The fause discovery rate (FDR) algorithm was used for adjusting *p* values. A *p* value < 0.05 was considered to be statistically significant. The correlation heatmap was created by pheatmap version 1.0.12. The intensity of dot colour represented the value of correlation coefficient which indicates the degree of association. The red and blue colours represented the positive and negatively correlations, respectively.

## Results

### The composition of gut fungal microbiota was changed in rats after long-term prednisone treatment

**Figure 1:**
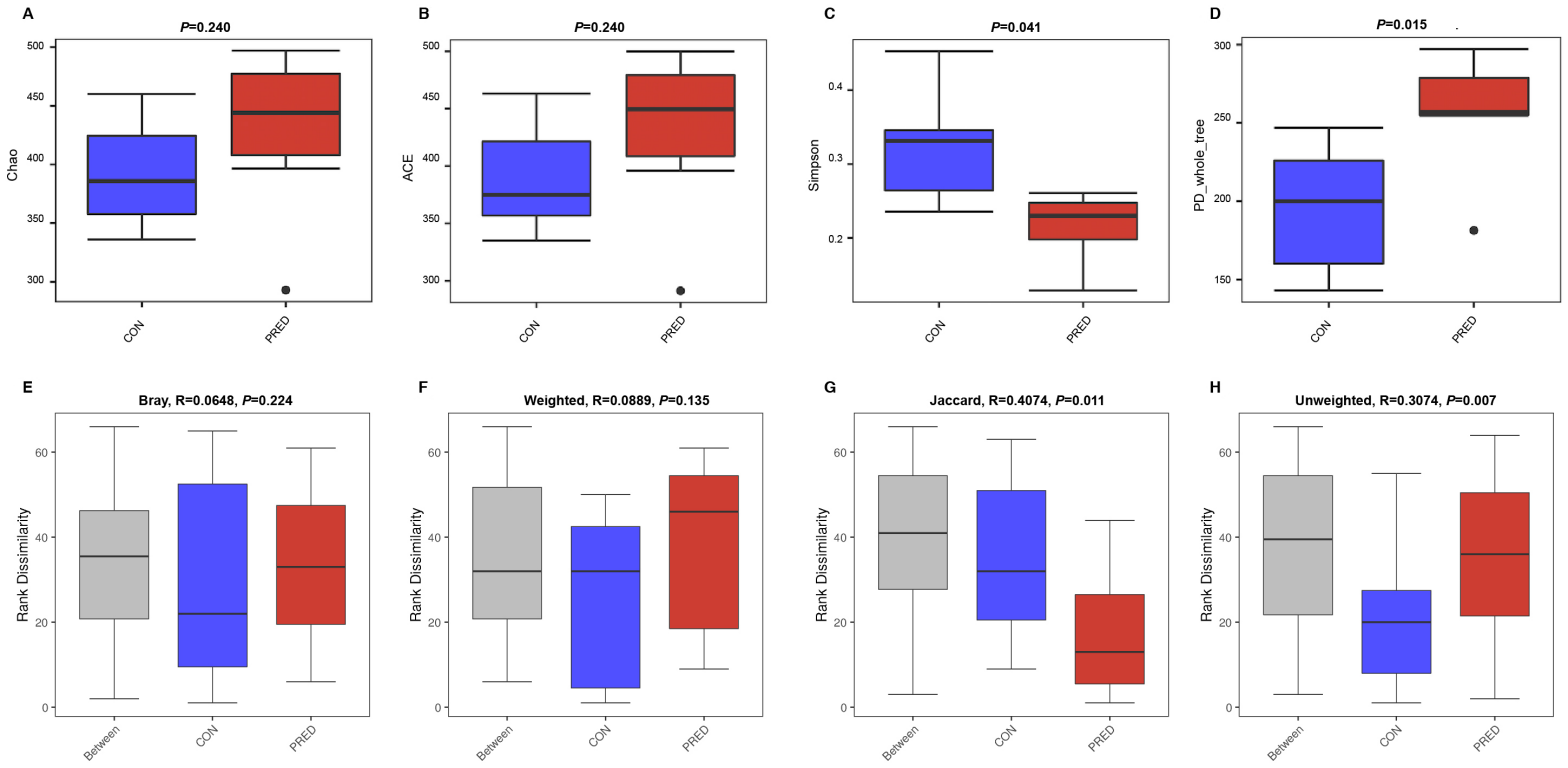
The alpha and beta diversity of gut mycobiome in rats after prednisone treatment. **(A)** and **(B)** The alpha diversity analysis of Chao and ACE indices between the CON and PRED groups showed that there were no changes in the richness of gut mycobiome in rats after prednisone treatment. CON versus PRED, *p* > 0.05. **(C)** and **(D)** There were significant differences in the alpha diversity of Simpson and PD_whole_tree indices, showing that the diversity of gut mycobiome increased significantly in rats treated with prednisone. CON versus PRED, ^*^p < 0.05. **(E)** and **(F)** The results of Bray–Curtis and weighted UniFrac distance showed that there were no significant differences between CON and PRED groups. **(G)** and **(H)** It was significantly separated between CON and PRED groups in Jaccard and Unweighted UniFrac distances, ^*^p < 0.05.

A total of 1,381 OTUs were detected in rat fecal samples (Supplementary Data-1). The richness and diversity of gut fungal microbiota in the CON and PRED groups were evaluated by using alpha diversity analysis including the Chao, ACE, Simpson and PD_whole_tree indices. The results showed that there were no changes in the richness of gut fungi in rats after prednisone treatment. However, the diversity of gut fungi increased significantly in rats treated with prednisone (Figure 1).

The difference in abundance distribution was analyzed by beta diversity analysis including Bray-Curtis, weighted UniFrac, Jaccard and unweighted UniFrac distances. The results of Bray-Curtis and Weighted UniFrac distance showed that there were no significant changes in the composition of gut fungal microbiota. However, it was significantly separated between the CON and PRED groups in the Jaccard and Unweighted UniFrac distances (Figure 1).

**Figure 2:**
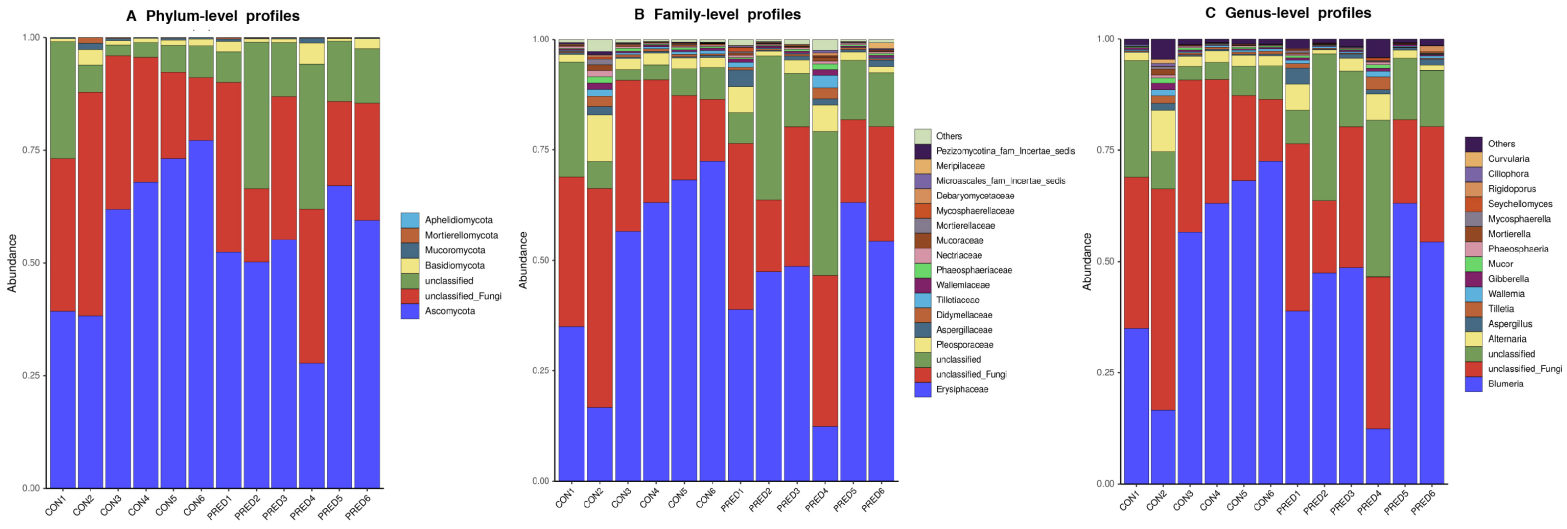
The compositional distribution of gut mycobiome in rats with and without prednisone treatment. The barplots of gut mycobiome showed the composition of enteric fungi at the phylum **(A)**, family **(B)**, and genus **(C)** levels. *Ascomycota* and *Erysiphaceae* were dominated at the phylum and family level, respectively. *Blumeria* was the most abundant fungal genus.

The composition of gut mycobiome in these fecal samples was dominated by *Ascomycota* and *Erysiphaceae* at the phylum and family level, respectively. The relative abundance of fungal genera in descending order was as follows: *Blumeria, unclassified Fungi*, *unclassified*, *Alternaria*, *Aspergillus*, *Tilletia*, *Wallemia*, *Gibberella*, *Mucor*, *Phaeosphaeria*, *Mortierella*, *Mycosphaerella*, *Seychellomyces*, *Rigidoporus*, *Ciliophora*, *Curvularia*, and others (Figure 2).

**Figure 3:**
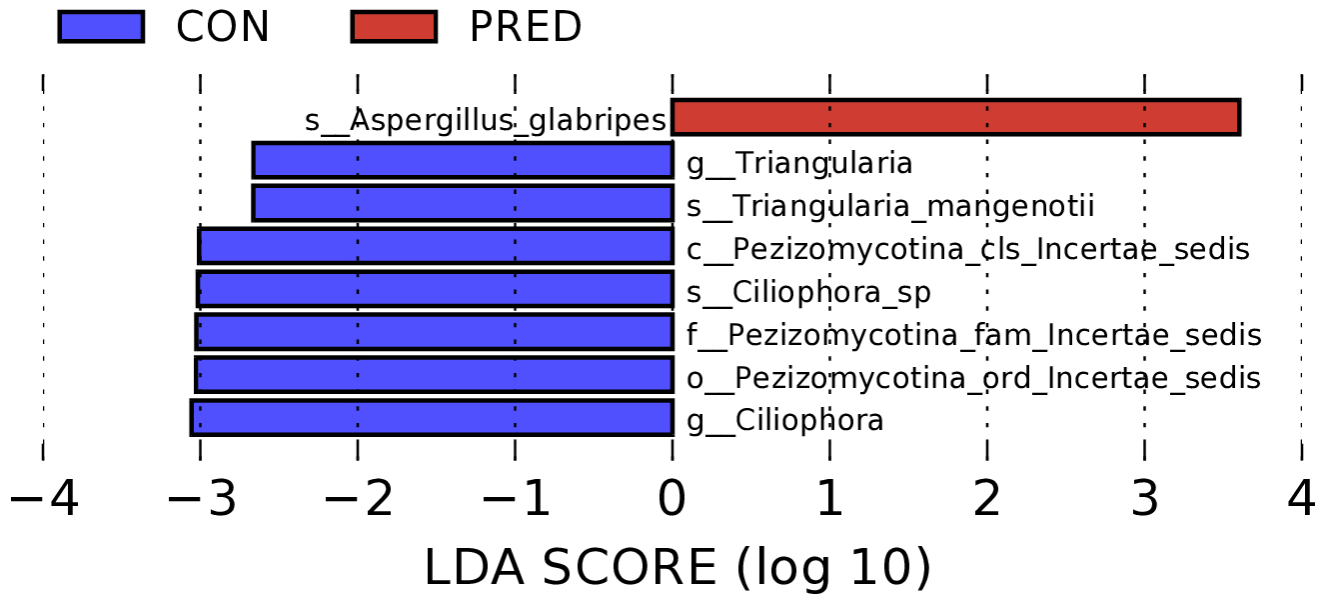
The differentially abundant gut fungi at taxonomic levels in rats after prednisone treatment. Linear discriminant analysis (LDA) effect size (LEfSe) analysis identified the differentially abundant enteric fungi at the class, order and family levels, respectively, (Kruskal–Wallis test, *p* < 0.05, LDA score > 2.0). The first lowercase letter preceding each fungal name represented the taxonomic level. c, class; o, order; f, family; g, genus; s, species.

As shown in the Figure 3, it revealed that prednisone decreased the relative abundance of *Pezizomycotina_cls_Incertae_sedis,Pezizomycotina_ord_Incertae_sedis*, *Pezizomycotina_fam_Incertae_sedis* at the class, order and family level, respectively. At the genus level, the relative abundance of *Triangularia* and *Ciliophora* decreased significantly in rats treated with prednisone for 6 weeks. In addition, at the species level, the relative abundance of *Aspergillus glabripes* increased significantly after prednisone treatment, while *Triangularia mangenotii* and *Ciliophora* sp.decreased significantly.

### The impact of prednisone treatment on the fungal intrakingdom interactions in rats

**Figure 4:**
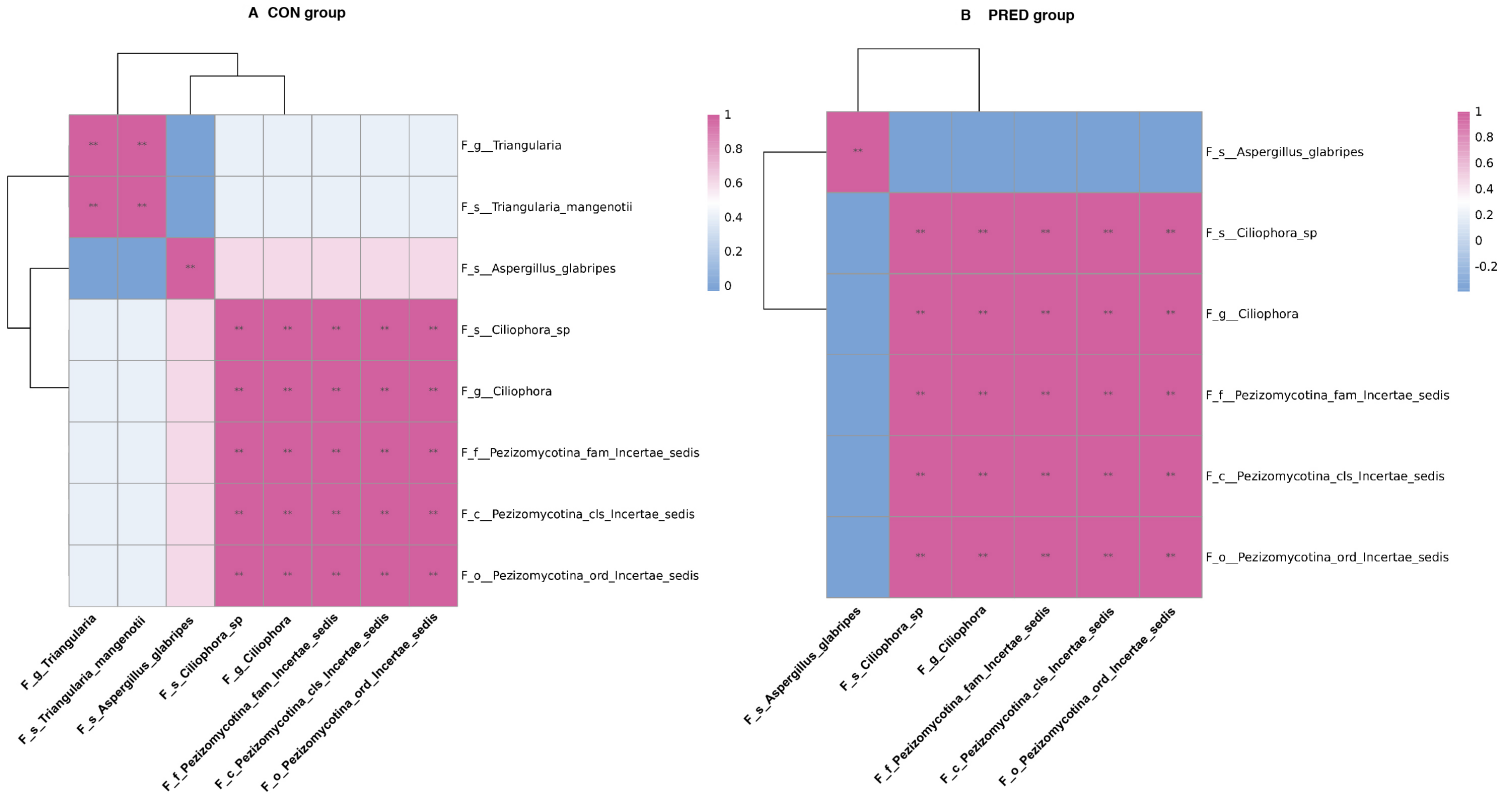
The impact of prednisone treatment on the fungal intrakingdom interactions in rats. The ecological interactions among differentially abundant fungi at taxonomic levels in CON group **(A)** and PRED group **(B)**. It showed that there were 30 and 26 intrakingdom correlations in CON group and PRED group, respectively. The red and blue colours represented the positive and negatively correlations, respectively. ^**^p < 0.01.

To investigate the potential ecological interactions within the group, the correlation analysis was performed between differentially abundant gut fungi at the taxonomic level. As shown in Figure 4, there were 30 and 26 intrakingdom correlations in the CON group and PRED group, respectively. The positive correlation between fungal genus *Triangularia* and species *Triangularia mangenotii* disappeared in rats after prednisone treatment. Since the species *Triangularia mangenotii* belongs to the genus *Triangularia*. Thus, changes in the fungal intrakingdom ecological interactions were not found in rats after prednisone treatment.

### The interkingdom interactions between differentially abundant gut fungi and bacteria in rats before and after prednisone treatment

**Figure 5:**
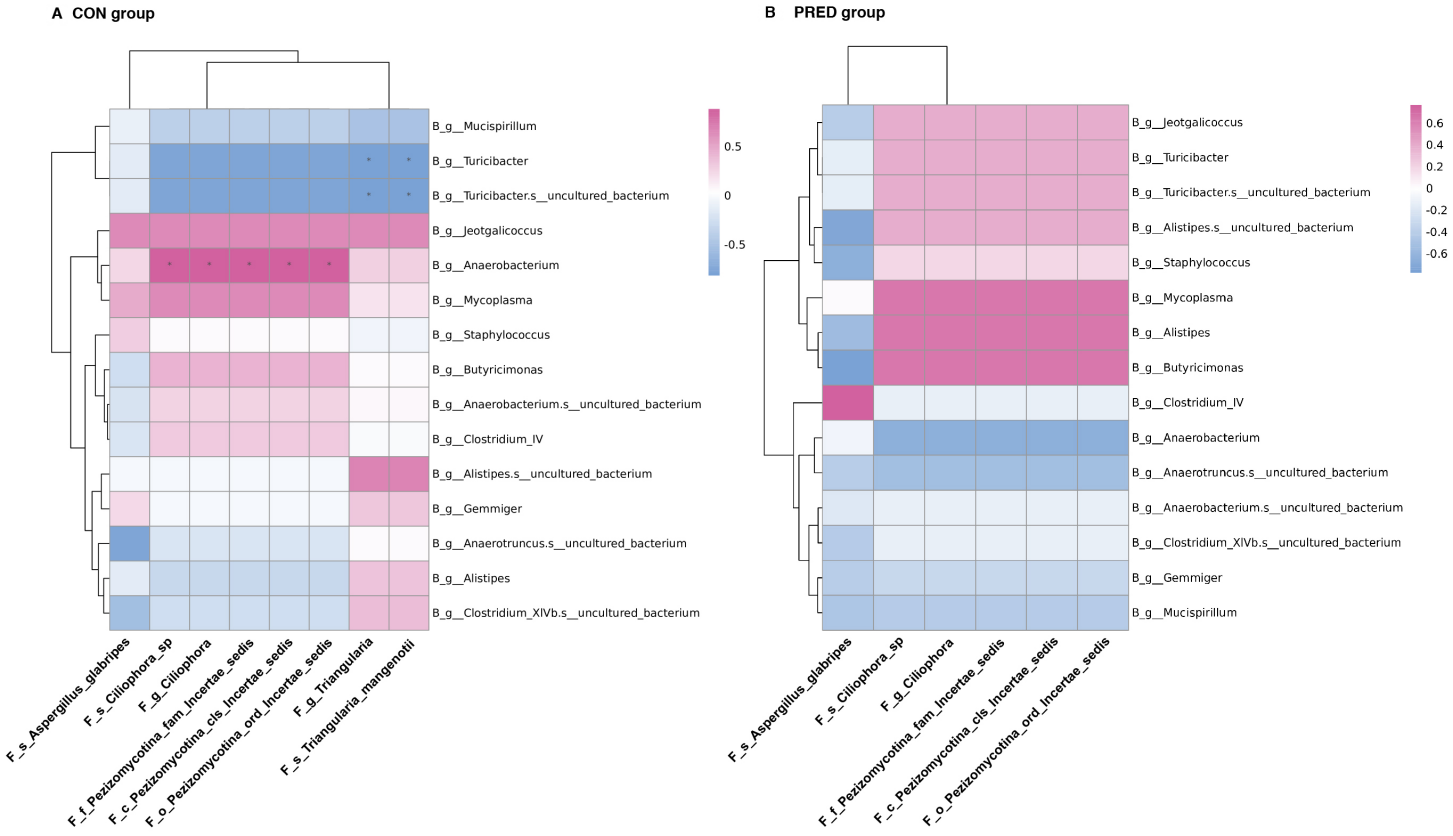
The ecological interactions between differentially abundant gut fungi and gut bacteria in rats before and after prednisone treatment. The interkingdom interactions between differentially abundant enteric fungi at taxonomic levels and gut bacteriome at the genus level in CON group **(A)** and PRED group **(B)**. The red and blue colours represented the positive and negatively correlations, respectively. ^*^p < 0.05.

In an attempt to determine the possible interkingdom interaction between gut fungi and bacteria before and after prednisone treatment, we performed correlation analysis between differentially abundant enteric fungi at the taxonomic level and gut bacteriome at the genus level. The data of differentially abundant gut bacteria were re-analysed from our previous research project (Zhang et al., 2021) for alignment with the used samples in this study (Supplementary Data-2). As shown in Figure 5, there were 9 and 0 interkingdom correlations in the CON and PRED groups, respectively. At the genus level, the fungus *Triangularia* was negatively correlated with the bacteria *Turicibacter* and *Turicibacter.s_uncultured_bacterium*, while the fungus *Ciliophora* was positively correlated with bacterium *Anaerobacterium*. However, these correlations disappeared in the rats after prednisone treatment.

### The correlations between differentially abundant gut fungi and fecal metabolites in rats

**Figure 6:**
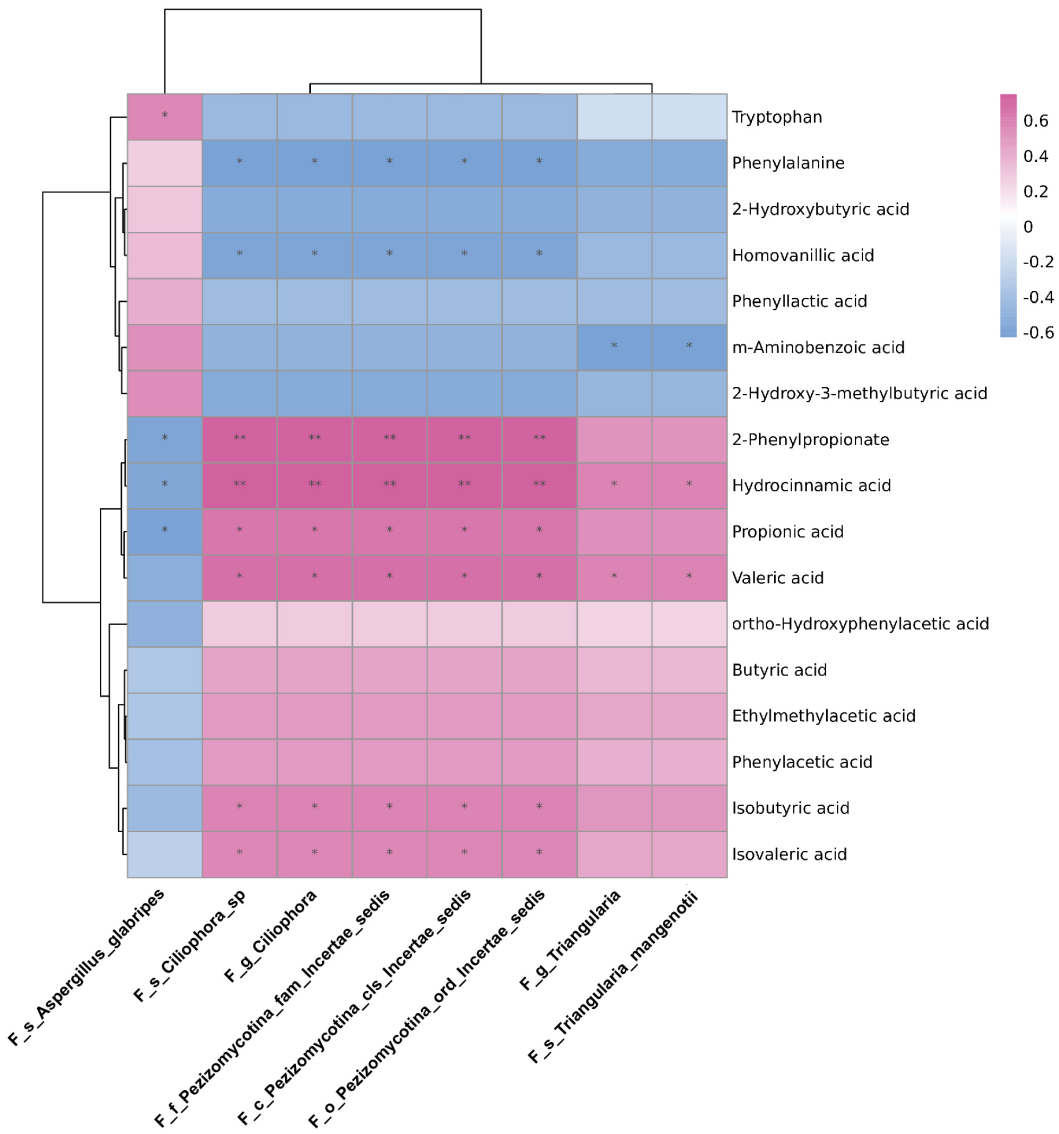
The correlations between differentially abundant gut fungi and fecal metabolites in rats. The correlations between differentially abundant gut fungi at taxonomic levels and fecal metabolites in rats. The intensity of dot colour represented the value of correlation coefficient which indicates the degree of association. The red and blue colours represented the positive and negatively correlations, respectively. ^*^p < 0.05 and ^**^p < 0.01.

To explore the possible resources of fecal metabolites, we analysed the correlations between differential abundant gut mycobiome and fecal metabolome. The data of differential fecal metabolites were re-analysed from our previous research project (Zhang et al., 2021; Supplementary Data-3). As shown in Figure 6, the genus *Triangularia* and species *Triangularia mangenotii* were negatively correlated with m-aminobenzoic acid, but positively correlated with hydrocinnamic acid and valeric acid. Another fungal genus *Ciliophora* and species *Ciliophora* sp. were negatively correlated with phenylalanine and homovanillic acid, but positively correlated with 2-Phenylpropionate, hydrocinnamic acid, propionic acid, valeric acid, isobutyric acid and isovaleric acid. The species *Aspergillus glabripes* was positively correlated with trytophan, but negatively correlated with 2-Phenylpropionate, hydrocinnamic acid and propionic acid.

## Discussion

Gut microbiota is a diverse ecological system which is dominated by enteric bacteria (Adak and Khan, 2019). Gut fungi only make up only a small proportion of gut microbiota, but they play an important role in maintaining host health (Begum et al., 2022). We are the first to show that the composition of gut fungi was changed after long-term prednisone treatment. Moreover, prednisone altered the fungi-bacteria interkingdom interactions in rats.

In this study, we found that the richness of enteric fungi was not changed significantly after 6 weeks of prednisone treatment, while the diversity of gut mycobiome increased. It suggested prednisone might promote the growth of certain fungi. In contrast, there are no changes in the richness and diversity of gut bacteria in rats after prednisone treatment in our previous study (Zhang et al., 2021). Taken together, prednisone was more effective in changing the composition of gut fungi than gut bacteria. Regarding the structure of fungal community in these rat fecal samples*, Ascomycota* and *Basidiomycota* accounted for the two largest proportions at the phylum level, with the exception of unclassified fungi. This finding is consistent with some previous reports showing that *Ascomycota* and *Basidiomycota* are the two dominant phyla in the digestive tract (Liguori et al., 2016; Richard and Sokol, 2019). Therefore, prednisone could change the composition of gut mycobiome in rats.

Among these changed fungus, the fungal species *Aspergillus glabripes* belongs to genus *Aspergillus* which is an opportunistic pathogen. The manifestations of *Aspergillus* infection varies from allergic to cavitary or angioinvasive syndromes depend on host immunological status (Chotirmall et al., 2014). It is known that the life-threatening *Aspergillus* infection can occur in immunocompromised patients (Wilmes et al., 2021). Long term treatment with GCs is a risk factor of *Aspergillus* infection (Gustafson et al., 1983). We found that the relative abundance of *Aspergillus glabripes* increased significantly after long-term prednisone treatment in rats. This finding suggested that gut fungi might be a resource of organ infection in patients receiving long-term GCs treatment. Our data also showed that *Triangularia mangenotii* and *Ciliophora* sp. decreased significantly after prednisone treatment. The fungal species *Triangularia mangenotii* belongs to be the phylum *Ascomycota*. It has been reported that the relative abundance of *Triangularia mangenotii* decreased in heavy metal Pb–Zn contaminated soil (Harms et al., 2021). However, *Triangularia mangenotii* and *Ciliophora sp*. have not been reported in human. The role of these three differentially abundant fungal species needs to be investigated in patients who received prednisone treatment in future studies.

Gut microbes could influence host immunity and pathophysiological processes by producing fecal metabolites. For instance, short chain fatty acids (SCFAs) serve as nutrients for enteric cells and are involved in shaping host immunity (Vinolo et al., 2011; Morrison and Preston, 2016). Our previous study showed that SCFAs including propionic acid, valeric acid, isobutyric acid and isovaleric acid decreased significantly after prednisone treatment. Furthermore, the decreased production of SCFAs was associated with decreased relative abundances of the bacterial genera *Alistipes* and *Clostridium XIVb* and increased *Anaerobacterium* (Zhang et al., 2021). In this study, correlation analysis revealed that the decreased fungal genus *Ciliophora* and species *Ciliophora sp.* might also contribute to the decreased production of SCFAs, including propionic acid, valeric acid, isobutyric acid and isovaleric acid. Additionally, the decreased relative abundance of fungal genus *Triangularia* might be associated with the decreased production of valeric acid. Similarly, hydrocinnamic acid was correlated with *Alistipes* in our previous study (Zhang et al., 2021), while it was correlated with *Triangularia* in this study. Therefore, the fecal metabolites might be the products of multiple gut microbes including bacteria and fungi. However, it remains to be verified which microorganisms play a major role in producing these metabolites in future studies.

Gut microbiota is a dynamic ecological system. Mutualistic and antagonistic relationships have been found among fungi or between fungi and bacteria (Tso et al., 2018; Rowan-Nash et al., 2019). We found that the fungal intrakingdom interaction in rats decreased after prednisone treatment, as the positive correlation between fungal genus *Triangularia* and species *Triangularia mangenotii* disappeared after prednisone treatment. Actually, species *Triangularia mangenotii* belongs to the genus *Triangularia*. Thus, there were no changes in the fungal intrakingdom ecological interactions in rats after prednisone treatment. However, we found that the interkingdom interactions between differentially abundant gut fungi and bacteria significantly decreased in rats after long-term prednisone treatment. The correlations between *Turicibacter* and *Triangularia, between Ciliophora and bacterium* disappeared after prednisone treatment. It suggested that prednisone might break these interkingdom associations. Even though these findings have not been reported in human, it has been known that gut fungi and bacteria can form polymicrobial biofilm which are involved in drug resistance and adaption to environmental conditions (Costa-Orlandi et al., 2017). For instance, *H.pylori* could survive in the vacuole of *Candida* spp. which protect against hostil gactric environment. This protection facilitates *H.pylori* to release toxins (Saniee et al., 2013). Theoretically, the reduced ecological interkingdom interactions may limit the growth of certain pathogens by disrupting the coculture platform. This might be associated with the anti-inflammatory effects of prednisone. Thus, uncovering the ecological microbial intra-and interkingdom interactions may help to understand the pharmacology of prednisone. However, these fungi-bacteria interaction results generating from statistical analysis needs be verified in future studies.

In conclusion, long-term prednisone treatment led to fungal microbiota dysbiosis and might alter the ecological interaction between gut mycobiome and bacteriome in rats. This study also had some limitations. As prednisone is widely used to treat immune disorders, the impact of these changed gut fungi on host immunity needs to be investigated. Furthermore, fungi are only a small part of the gut microbiota, but this may be an underestimate. For instance, some fungi have not been annotated and classified, as the fungal genome database is incomplete and unable to annotate all fungi. Furthermore, the interactions between gut mycobiome and gut bacteriome/fecal metabolites need to be verified in future studies, as it will help to improve our understanding of the pharmacology of GCs and also explore therapeutic strategies for immune-mediated diseases.

## Data availability statement

The data presented in the study are deposited in the Sequence Read Archive (SRA) at NCBI, accession number: PRJNA 925437.

## Ethics statement

The animal study was reviewed and approved by Ethical Committee of Shanghai Children’s Hospital, School of Medicine, Shanghai Jiao Tong University.

## Author contributions

YK was responsible for the design of the study, analysing data, and manuscript writing. LS participated in analysing data and revising manuscript. WL and YS participated in experiments and manuscript writing. JZ participated in experiments. MW, G-hZ, and W-yH participated in analysing data. All authors contributed to the article and approved the submitted version.

## Funding

This study was financially supported by grants from Science and Technology Commission of Shanghai Municipality (21Y11904400) and clinical research grant of Shanghai Children’s Hospital (2020YLYZ02).

## Conflict of interest

The authors declare that the research was conducted in the absence of any commercial or financial relationships that could be construed as a potential conflict of interest.

The reviewer CL declared a shared affiliation with the authors to the handling editor at the time of the review.

## Publisher’s note

All claims expressed in this article are solely those of the authors and do not necessarily represent those of their affiliated organizations, or those of the publisher, the editors and the reviewers. Any product that may be evaluated in this article, or claim that may be made by its manufacturer, is not guaranteed or endorsed by the publisher.
